# Supplementary material for: Mediating factors in the relationship between combat-related traumatic injury and myocardial blood flow reserve: The ADVANCE cohort study
Source: PLoS One. 2026 Mar 19;21(3):e0345019. doi: 10.1371/journal.pone.0345019 (PMC13001918; doi:10.1371/journal.pone.0345019)
Supplement: S1 Table — (DOCX) [file pone.0345019.s001.docx]

**Table E1:** Correlations (ρ) between mediators

|  | VFM | Hs-CRP | 6MWD | RMMSD |  |  | |  |  |  |
| --- | --- | --- | --- | --- | --- | --- | --- | --- | --- | --- |
| VFM | - |  |  |  |  |  | |  |  |  |
| HSCRP | 0.357 | - |  |  |  |  | |  |  |  |
| 6MWD | -0.130 | -0.160 | - |  |  |  | |  |  |  |
| RMMSD | -0.264 | -0.086 | 0.096 | - |  |  | |  |  |  |
| Hs-CRP, high-sensitivity C reactive protein; RMSSD root mean square of successive differences; 6MWD, six-minute walk distance | | | | | | |  |  |  |  |
