# Supplementary material for: Mediating factors in the relationship between combat-related traumatic injury and myocardial blood flow reserve: The ADVANCE cohort study
Source: PLoS One. 2026 Mar 19;21(3):e0345019. doi: 10.1371/journal.pone.0345019 (PMC13001918; doi:10.1371/journal.pone.0345019)
Supplement: Attachment — Submitted filename: Review.pdf [file pone.0345019.s002.pdf]

Summary:

This manuscript entitled *"Mediating Factors in the Relationship between Combat-related Traumatic Injury and Myocardial Blood Flow Reserve: the ADVANCE Cohort Study"* investigates whether specific cardiovascular risk factors mediate the relationship between combat-related traumatic injury (CRTI) and myocardial blood flow reserve, represented by the subendocardial viability ratio (SEVR), using longitudinal data from the ADVANCE cohort. The study leverages a valuable dataset of UK servicemen (n=1,018, 504 with CRTI, of whom 140 were amputees, and 514 uninjured men, frequency-matched, by age, rank and deployment) with a follow-up duration for more than a decade after deployment. From the standpoint of public health significance, innovation, and scientific contribution, this paper represents a substantive and meaningful addition to the emerging literature on the long-term cardiovascular consequences of military trauma. The manuscript is suitable for publication pending modifications and clarifications described below:

Significance and Impact

The primary strength of this work lies in addressing an understudied question with substantial clinical implications: why combat-injured veterans exhibit elevated cardiovascular risk even years after injury. While prior papers from the same ADVANCE cohort established associations between CRTI, adverse cardiometabolic profiles, and reduced SEVR, this study advances the field by investigating the underlying mechanisms. Understanding modifiable mediators such as visceral fat mass, heart-rate variability, and physical function is crucial for designing targeted interventions and informing long-term rehabilitation strategies for injured service members. Given the large population of veterans with blast injuries, limb loss, and related trauma, these insights could have high translational relevance for both defense health systems and civilian trauma rehabilitation.

In terms of the approach, this study's main novelty lies in its rigorous application of counterfactual mediation analysis. Prior literature linking traumatic injury to cardiovascular risk has largely been retrospective, cross-sectional, or limited to mortality outcomes. By contrast, to strengthen causal inference, this manuscript uses longitudinal measurements: baseline (~8 years post-injury) for mediators and follow-up (~3 years later) for SEVR. The approach allows the authors to quantify the extent to which specific physiological domains (autonomic balance, adiposity, inflammation, physical activity, and physical function) explain the CRTI–SEVR association. The subgroup analysis focusing on traumatic amputation is particularly innovative and important, offering mechanistic hypotheses tailored to a clinical subgroup known to be at heightened cardiovascular risk.

Major comments:

- As mentioned above, beyond the scientific findings put forth in this manuscript, there are several potentially translational findings, such as designing interventions focused on HR variability or visceral fat. The authors are encouraged to explore this aspect further and provide additional insight into possibilities for future clinical studies.
- Although the manuscript acknowledges its limitation in unmeasured mediator-outcome confounding, the implication is potentially substantial. Variables such as resting heart rate, blood pressure, lipid profile, pain severity, mental health symptoms, medication use, and

sleep quality are likely to influence both the mediators (e.g., HRV, physical function) and SEVR. Without addressing or adjusting for these, the causal interpretations of mediation effects remain tentative. Could the authors perform sensitivity or bias analyses to quantify the strength of this confounding?

- Despite the decent cohort size (n=1,018) and high retention rate (~89%) large proportion of the data is considered missing (especially the ~25% missing RMSSD values). The missing HRV data in particular could introduce significant bias, especially if missingness differs systematically between injured and uninjured participants. This reviewer recommends performing a multiple imputation analysis, compare imputed vs. complete-case results, and provide missing-data patterns (e.g., missing by subgroup).
- Lack of clear justification for treating mediators independently: The mediators (VFM, HRV, 6MWD, LT-MVPA, Hs-CRP) are physiologically interrelated. Testing them one at a time oversimplifies the true causal structure and risks misleading conclusions. It would make better sense to perform sequential or joint mediation models.
- The reduction in SEVR is statistically significant, but the clinical significance for this demographic (mostly young men) is not clearly contextualized. It would be helpful if the authors compared observed SEVR differences to established risk thresholds or known effect sizes in metabolic syndrome, hypertension, or coronary disease cohorts.

Minor comments:

- The terms “baseline” and “eight years post-injury” could be defined more clearly. A visual description could also be included in figure 1.
- Figure 1 could be improved in quality and descriptiveness.
- I suggest superimposing the scatter plot on figure 2 for better visualization.
