# Supplementary material for: Mediating factors in the relationship between combat-related traumatic injury and myocardial blood flow reserve: The ADVANCE cohort study
Source: PLoS One. 2026 Mar 19;21(3):e0345019. doi: 10.1371/journal.pone.0345019 (PMC13001918; doi:10.1371/journal.pone.0345019)
Supplement: Attachment — Submitted filename: Final Rebutal 1 to SEVR Manuscript Plos One.docx [file pone.0345019.s003.docx]

Dear Professor Pahlevan

‘Mediating Factors in the Relationship between Combat-related Traumatic Injury and Myocardial Blood Flow Reserve: the ADVANCE Cohort Study’

ONE-D-25-50727

We would like to sincerely thank the three reviewers for taking the time to examine our manuscript and to provide such comprehensive reviews. We have responded (R) to each of the comments raised. We feel that we have been able to address all of the comments raised. We have, as requested also included both a tracked changes file and one without showing the tracked changes. We hope that our manuscript might now be considered suitable for publication in PLOS One.

“This study is a project by the ADVANCE study (grant number: ADV-ADMR-03), which is funded through the ADVANCE Charity. Key contributors to the charity are the Headley Court Charity (principal funder), HM Treasury (LIBOR Grant), Help for Heroes, Nuffield Trust for the Forces of the Crown, Forces in Mind Trust, National Lottery Community Fund, Blesma - The Limbless Veterans, the UK Ministry of Defence, and the Office for Veterans’ Affairs (OVA). The funders of the study had no role in study design, data collection, data analysis, data interpretation, or writing of the manuscript.”

We note that one or more of the authors is affiliated with the funding organization, indicating the funder may have had some role in the design, data collection, analysis or preparation of your manuscript for publication; in other words, the funder played an indirect role through the participation of the co-authors.

If the funding organization did not play a role in the study design, data collection and analysis, decision to publish, or preparation of the manuscript and only provided financial support in the form of authors' salaries and/or research materials, please review your statements relating to the author contributions, and ensure you have specifically and accurately indicated the role(s) that these authors had in your study in the Author Contributions section of the online submission form. Please make any necessary amendments directly within this section of the online submission form.  Please also update your Funding Statement to include the following statement: “The funder provided support in the form of salaries for authors [insert relevant initials], but did not have any additional role in the study design, data collection and analysis, decision to publish, or preparation of the manuscript. The specific roles of these authors are articulated in the ‘author contributions’ section.”

R: *The funding organisation did not play a role in the study design, data collection and analysis, decision to publish, or preparation of the manuscript.*

If the funding organization did have an additional role, please state and explain that role within your Funding Statement.

Please also provide an updated Competing Interests Statement declaring this commercial affiliation along with any other relevant declarations relating to employment, consultancy, patents, products in development, or marketed products, etc.

Within your Competing Interests Statement, please confirm that this commercial affiliation does not alter your adherence to all PLOS ONE policies on sharing data and materials by including the following statement: "This does not alter our adherence to  PLOS ONE policies on sharing data and materials.” (as detailed online in our guide for authors http://journals.plos.org/plosone/s/competing-interests). If this adherence statement is not accurate and there are restrictions on sharing of data and/or materials, please state these. Please note that we cannot proceed with consideration of your article until this information has been declared.

*R: This has been updated*

3. We note that you have indicated that there are restrictions to data sharing for this study. For studies involving human research participant data or other sensitive data, we encourage authors to share de-identified or anonymized data. However, when data cannot be publicly shared for ethical reasons, we allow authors to make their data sets available upon request. For information on unacceptable data access restrictions, please see http://journals.plos.org/plosone/s/data-availability#loc-unacceptable-data-access-restrictions.

Before we proceed with your manuscript, please address the following prompts:

a) If there are ethical or legal restrictions on sharing a de-identified data set, please explain them in detail (e.g., data contain potentially identifying or sensitive patient information, data are owned by a third-party organization, etc.) and who has imposed them (e.g., a Research Ethics Committee or Institutional Review Board, etc.). Please also provide contact information for a data access committee, ethics committee, or other institutional body to which data requests may be sent.

R. *The data underlying this study are not publicly available due to ethical and legal restrictions. Although the dataset has been de-identified, it contains sensitive personal and clinical information. The combination of these variables poses a non-trivial risk of participant re-identification, particularly for individuals in small or vulnerable subgroups. For this reason, unrestricted public sharing of the data could compromise participant confidentiality.*

These restrictions were imposed by the [*Project Board??*] to protect participant confidentiality. Data may be made available upon reasonable request to qualified researchers, subject to approval by the relevant committee and the implementation of appropriate data protection safeguards. Requests should be directed to [*ADVANCE study Data Team??*] at [­­*adv_data_team@imperial.ac.uk*].

b) If there are no restrictions, please upload the minimal anonymized data set necessary to replicate your study findings to a stable, public repository and provide us with the relevant URLs, DOIs, or accession numbers. Please see http://www.bmj.com/content/340/bmj.c181.long for guidelines on how to de-identify and prepare clinical data for publication. For a list of recommended repositories, please see https://journals.plos.org/plosone/s/recommended-repositories. You also have the option of uploading the data as Supporting Information files, but we would recommend depositing data directly to a data repository if possible.

Please update your Data Availability statement in the submission form accordingly.

4. Please note that your Data Availability Statement is currently missing [the repository name and/or the DOI/accession number of each dataset OR a direct link to access each database]. If your manuscript is accepted for publication, you will be asked to provide these details on a very short timeline. We therefore suggest that you provide this information now, though we will not hold up the peer review process if you are unable.

*R :Please see above*

5. Please be informed that funding information should not appear in the Acknowledgments section or other areas of your manuscript. We will only publish funding information present in the Funding Statement section of the online submission form. Please remove any funding-related text from the manuscript.

R: *The funding information has been removed from the acknowledgements section*

6. Your ethics statement should only appear in the Methods section of your manuscript. If your ethics statement is written in any section besides the Methods, please delete it from any other section.

*R: The ethics statement is now only in the methods section*

7. We note you have included a table to which you do not refer in the text of your manuscript. Please ensure that you refer to Table 4 in your text; if accepted, production will need this reference to link the reader to the Table.

8. Please include captions for your Supporting Information files at the end of your manuscript, and update any in-text citations to match accordingly. Please see our Supporting Information guidelines for more information: http://journals.plos.org/plosone/s/supporting-information.

9. If the reviewer comments include a recommendation to cite specific previously published works, please review and evaluate these publications to determine whether they are relevant and should be cited. There is no requirement to cite these works unless the editor has indicated otherwise.

R: *This has been done*

10. Please review your reference list to ensure that it is complete and correct. If you have cited papers that have been retracted, please include the rationale for doing so in the manuscript text, or remove these references and replace them with relevant current references. Any changes to the reference list should be mentioned in the rebuttal letter that accompanies your revised manuscript. If you need to cite a retracted article, indicate the article’s retracted status in the References list and also include a citation and full reference for the retraction notice.

R: *This has been done*


The [PLOS Data policy](https://gbr01.safelinks.protection.outlook.com/?url=http%3A%2F%2Ftrack.editorialmanager.com%2FCL0%2Fhttp%3A%252F%252Fwww.plosone.org%252Fstatic%252Fpolicies.action%2523sharing%2F1%2F010f019a9ff67adb-424c3e26-5f40-48b3-96da-d811173fe6ec-000000%2FLfqLpR0GKD9aZyjSiRK8R3bpLdOZlUkq_Rq2vNl9-CI%3D238&data=05%7C02%7Cchristopher.boos%40nhs.net%7Cafe9c00a151d4d4ff3d008de284f37cc%7C37c354b285b047f5b22207b48d774ee3%7C0%7C1%7C638992518142411432%7CUnknown%7CTWFpbGZsb3d8eyJFbXB0eU1hcGkiOnRydWUsIlYiOiIwLjAuMDAwMCIsIlAiOiJXaW4zMiIsIkFOIjoiTWFpbCIsIldUIjoyfQ%3D%3D%7C0%7C%7C%7C&sdata=AXGlHdiFd9FR5oz%2FcObJ01MLqi9DBcwId%2FuvSZtZVos%3D&reserved=0) requires authors to make all data underlying the findings described in their manuscript fully available without restriction, with rare exception (please refer to the Data Availability Statement in the manuscript PDF file). The data should be provided as part of the manuscript or its supporting information, or deposited to a public repository. For example, in addition to summary statistics, the data points behind means, medians and variance measures should be available. If there are restrictions on publicly sharing data—e.g. participant privacy or use of data from a third party—those must be specified.

Reviewer #1: Yes

Reviewer #2: No

Reviewer #3: Yes

Reviewer #1: This is an original paper that examines the relationship between certain cardiovascular risk factors including visceral fat, heart rate variability, physical functioning in patients with combat related traumatic injury or without injury or amputation and subendocardial viability ratio. They find reduced SEVR in injured amputees and other fascinating associations. Some of this seems like common sense. For example, an amputee is not going to be able to necessarily exercise in the same fashion as a non injured amputee and therefore coronary reserve is likely to be better in the non injured individual. My major criticism is that we are not provided information about other important cardiovascular risk factors here: Cholesterol, LDL, HDL, triglyceride values; information on diabetes, Hemoglobin A1C, hypertension, medicines.

*R. Thank you for raising this very important point. In our study we examined the examined the mediating role of cardiovascular risk factors, shown to significantly greater with CRTI at study baseline (~8 years post-injury/deployment) on SEVR, measured three-years later (FU1).*

*There have been several Cardiovascular manuscripts that have been published in relation to the baseline data collection from the full ADVANCE (n=1144) Cohort. And design and protocol of the ADVANCE study have been published and cited in our current manuscript.*

*These Cardiovascular manuscripts have examined an extremely wide range of cardiovascular risk factors among the full cohort of 579 male adult UK combat veterans (UK-Afghanistan War 2003-2014) with combat injury CRTI who were frequency-matched to 565 uninjured men by age, service, rank, regiment, deployment period and role-in-theatre. Our baseline publications have also examined the Influence of injury type (amputation), severity (Injury severity scores) and mechanism (blast etc). The comparative risk factors examined at baseline (between the injured and uninjured) have included measures of inflammation (Hs-CRP, neutrophil-lymphocyte ratio), Arterial stiffness (arterial augmentation index and pulse wave velocity), Cardiometabolic health (metabolic syndrome), glycaemic status (fasting glucose and HbA1C), obesity (BMI, abdominal waist circumference), physical activity (using the IPAQ questionnaire) and physical function (6MWD) measures of visceral fat (using DEXA), fasting blood lipid profiles, cardiovascular proteomics (in submission), heart rate variability (HRV) and non-invasive measurement of myocardial blood flow (SEVR using the Vicorder device) as well as the comparative global cardiovascular risk prediction scores using the 21-item QRISK III Scoring tool. We have also examined the influence of a variety of metal health assessments including PTSD, anxiety, depression and sleep on various measures of cardiovascular risk including HRV (measured at baseline). Of note the SEVR was significantly lower in the injured vs uninjured group at baseline and even lower in those with more severe injuries.*

*Several examples of these publications in relations to the baseline data collection for ADVANCE are displayed below.*

1. *Boos CJ, Schofield S, Cullinan P, Dyball D, Fear NT, Bull AMJ, Pernet D, Bennett AN; ADVANCE study. Association between combat-related traumatic injury and cardiovascular risk. Heart. 2022 Mar;108(5):367-374. doi: 10.1136/heartjnl-2021-320296. Epub 2021 Nov 25. PMID: 34824088; PMCID: PMC8862100.*
2. *Boos CJ, Schofield S, Bull AMJ, Fear NT, Cullinan P, Bennett AN; ADVANCE Study. The relationship between combat-related traumatic amputation and subclinical cardiovascular risk. Int J Cardiol. 2023 Nov 1;390:131227. doi: 10.1016/j.ijcard.2023.131227. Epub 2023 Jul 30. PMID: 37527753.*
3. *Boos CJ, Haling U, Schofield S, Cullinan P, Bull AMJ, Fear NT, Bennett AN; ADVANCE Study. Relationship between combat-related traumatic injury and its severity to predicted cardiovascular disease risk: ADVANCE cohort study. BMC Cardiovasc Disord. 2023 Nov 27;23(1):581. doi: 10.1186/s12872-023-03605-0. PMID: 38012542; PMCID: PMC10680223.*
4. *Maqsood R, Schofield S, Bennett AN, Bull AM, Fear NT, Cullinan P, Khattab A, Boos CJ; ADVANCE study. Relationship between combat-related traumatic injury and ultrashort term heart rate variability in a UK military cohort: findings from the ADVANCE study. BMJ Mil Health. 2024 Dec 11;170(e2):e122-e127. doi: 10.1136/military-2022-002316. PMID: 36990509; PMCID: PMC11672064.*
5. *Dyball D, Bennett AN, Schofield S, Cullinan P, Boos CJ, Bull AMJ, Stevelink SA, Fear NT;*

*ADVANCE Study. The underlying mechanisms by which PTSD symptoms are associated with cardiovascular health in male UK military personnel: The ADVANCE cohort study. J Psychiatr Res. 2023 Mar;159:87-96. doi: 10.1016/j.jpsychires.2023.01.010. Epub 2023 Jan 17. PMID: 36696788.*

6. *Maqsood R, Schofield S, Bennett AN, Khattab A, Clark C, Bull AMJ, Fear NT, Boos CJ. The Influence of Physical and Mental Health Mediators on the Relationship Between Combat-Related Traumatic Injury and Ultra-Short-Term Heart Rate Variability in a U.K. Military Cohort: A Structural Equation Modeling Approach. Mil Med. 2024 Feb 27;189(3-4):e758-e765. doi: 10.1093/milmed/usad341. PMID: 37656495; PMCID: PMC10898941.*
7. *Maqsood R, Schofield S, Bennett AN, Khattab A, Bull AMJ, Fear NT, Cullinan P, Boos CJ. The inverse and non-linear association between central augmentation index and heart rate variability in a cohort of male British combat personnel- findings from the ADVANCE study. Blood Press. 2025 Dec;34(1):2524409. doi: 10.1080/08037051.2025.2524409. Epub 2025 Jul 10. PMID: 40549417.*

*Hence, it would have been inappropriate and repetitious to examine many of these cardiovascular risk markers again among the injured and uninjured at baseline. Moreover, for this current study we examined only the participants who went on to have SEVR measured again at follow up 1 three years later (approximately 11 years post injury/deployment) so that we could undertake a robust mediation model to investigate several of the potential factors that might explain the lower SEVR among the injured. This would allow us to potentially infer causation rather than simple association.*

For the HRV measures how long was the pulse or ECG measured?

R*. We had referenced the full details and methodology of the baseline HRV measurement in the manuscript. Essentially, this consisted of an ultrashort recording period of approximately 14 seconds. The calculation of the inter-beat interval to quantify HRV was obtained from arterial waveform data captured and stored during the measurement of pulse waveform velocity using the Vicorder device. We had full disclosure of the waveforms and any erroneous waveforms within the 14 second sequence were manually excluded during HRV signal processing in Kubios. This waveform HRV measurement method has been previously validated and published for our cohort. At the first follow up (follow up 1) for ADVANCE we introduced single lead ECG-recording in addition to the ongoing use of the Vicorder device to obtain pulse waveform analyses, arterial stiffness and central blood pressure measurements. Hence from follow up onwards (not baseline) we have the ability to measure HRV to gold standard using a continuous ECG method over 5-minute epochs. Nevertheless, we have found a strong agreement between the two methods of measuring HRV and this work has been* published:

*Maqsood R, Schofield S, Bennett AN, Khattab A, Bull AMJ, Fear NT, Boos CJ. Validity of Ultra-Short-Term Heart Rate Variability Derived from Femoral Arterial Pulse Waveform in a British Military Cohort. Appl Psychophysiol Biofeedback. 2024 Dec;49(4):619-627. doi: 10.1007/s10484-024-09652-3. Epub 2024 Jul 11. PMID: 38990252; PMCID: PMC11588943*.

*In the light of your comments we have updated the methods in our paper to make it clearer as to how HRV was measured and processed.*

How were arrhythmias handled?

*Thank you for raising this important issue. As HRV was measured at baseline using pulse waveform analysis and inter-beat interval (not RR interval-ECG) data we could not directly identify arrhythmias. The capacity to measure HRV using ECG data only became available at Follow up 1 onwards. However, we have full disclosure of the arterial waveform data and where there was clear evidence of extreme shortening of the consecutive arterial waveforms to suggest an ectopic with differing amplitudes (which were extremely rare) suggestive of potential ectopy this segment was voided from the HRV analysis as they were flagged in the Kubios HRB analysis software. Examination of the complete ECG data from FU1 have shown that the data is of high quality with no sustained arrhythmias seen.*

Data on depression/anxiety and emotional stress are not provided, but it is well known that psychological stress can contribute to cardiovascular events. What medicines were the participants on .... were any on vasodilators including things like calcium channel blockers, nitrates, PDE5 inhibitors... Viagra like drugs, since these can all cause vasodilation including of the coronary arteries. There should be some discussions related to these issues or data provided if available on medicines.

*R. Thank you for raising this very important point. The mental health outcomes for the full baseline ADVANCE Cohort have been published.*

*Dyball D, Bennett AN, Schofield S, Cullinan P, Boos CJ, Bull AMJ, Wessely S, Stevelink SAM, Fear NT; ADVANCE study. Mental health outcomes of male UK military personnel deployed to Afghanistan and the role of combat injury: analysis of baseline data from the ADVANCE cohort study. Lancet Psychiatry. 2022 Jul;9(7):547-554. doi: 10.1016/S2215-0366(22)00112-2. PMID: 35717965.*

*It was shown that whilst the injured had significantly worse mental health outcomes than the uninjured this was mainly driven by the injured non-amputees. The amputees actually had significantly lower anxiety, depression and PTSDs scores than the injured non amputees. Overall, sustaining a combat injury was associated with a 46–67% increase in odds of reporting PTSD, depression, and anxiety symptoms compared with uninjured personnel. Planned subgroup analysis results had shown these differences were driven mostly by those with non-amputation injuries, and that individuals who had amputation injuries had minimal differences in the odds of reporting probable PTSD, anxiety, or depression compared with uninjured personnel and notably lower odds of reporting poor mental health outcomes compared with individuals who had non-amputation-related injuries.*

*The primary outcome of interest in this current study is the subendocardial viability ratio (SEVR). An association between PTSD and mental health outcomes and SEVR has not been previously demonstrated. Hence, we aimed to investigate mediators that have been shown previously and form our baseline ADVANCE papers to be associated with the SEVR and potentially causally related.*

*Our group have also published a follow up study from the baseline ADVANCE cohort in which the relationship between PTSD clusters and cardiovascular risk were examined. In this second manuscript (see below) no associations were observed between PTSD symptom clusters and high sensitivity c-reactive protein, diastolic BP, total cholesterol or fasting glucose. PTSD were associated with increased cardiovascular risk via cardiometabolic (visceral fat and triglycerides) and haemodynamic functioning (heart rate) mechanisms, but not inflammation*. Hence it is not unreasonable to hypothesise that PTSD could influence SEVR. However, this well beyond the scope of the current study and as previously mentioned PTSD was not worsened by amputee status.

*Dyball D, Bennett AN, Schofield S, Cullinan P, Boos CJ, Bull AMJ, Stevelink SA, Fear NT; ADVANCE Study. The underlying mechanisms by which PTSD symptoms are associated with cardiovascular health in male UK military personnel: The ADVANCE cohort study. J Psychiatr Res. 2023 Mar;159:87-96. doi: 10.1016/j.jpsychires.2023.01.010. Epub 2023 Jan 17. PMID: 36696788.*

Reviewer #2: The authors present a valuable analysis using the ADVANCE cohort to investigate the mediating pathways linking combat-related traumatic injury (CRTI) to long-term reductions in myocardial blood flow reserve (MBFR), as measured by the Subendocardial Viability Ratio (SEVR). The study addresses a clinically important question regarding the long-term cardiovascular health of service personnel. The use of a robust, prospective cohort and the application of mediation analysis are strong methodological points. The manuscript is generally well-structured. However, several critical details concerning the mediation model's reporting, methodology, and interpretation need to be fully clarified and addressed before moving forward.

Major Comments:
1- The core methodology of this manuscript is mediation analysis. It is crucial to report the analysis with explicit detail. Given that the cohort was frequency-matched (by age, rank, and deployment), please detail how these matching factors were included in the mediation models (as covariates or via a more specialized approach).

*R. Apologies if this was not clear. We have now amended to text to show that age and rank were included as confounders in the mediation model. As the cohort was frequency matched on the exposure, no specialized methods were required.*

*“Confounders (age at injury/deployment and rank) of the exposure-mediator and mediator-outcome relationships were included in the models.”*

2- The results must clearly state the full statistical picture of the mediation. What was the calculated Indirect Effect (IE) through the identified mediator(s)? Explicitly state whether the finding represents full mediation (CDE non-significant) or partial mediation (CDE significant). This is a required step for interpreting mediation models.

*R. Thank you for your comment. We state the effect size and CI of the Natural Indirect Effect (NIE) for each mediator in the table. In the text we refer to the NIE, however we do focus more on the CDE and proportion eliminated due to the more relaxed assumptions of these and we do discuss the possible bias of the NIE due to possible exposure-induced confounders.*

*Discussing mediation in terms of full and partial was discussed, however more recently many authors have advised against this terminology.*

*Memon, Mumtaz & Cheah, Jun-Hwa & Ramayah, T. & Ting, Hiram & Chuah, Francis. (2018). Mediation Analysis: Issues and Recommendations. Journal of Applied Structural Equation Modeling. 2. i-ix. 10.47263/JASEM.2(1)01.*

*If we state that we have full mediation, it may also imply that we have explained the association between exposure and outcome. This of course would be misleading, especially as we have addressed our limitations of exposure-induced confounders. Therefore, rather than using the terms full and partial mediation, we provide the effects sizes and confidence intervals of the NIE, NDE and TE for the reader to draw conclusions.*

3- The legend for Figure 2 mentions an exposure-mediator interaction. If this interaction term was statistically significant, the standard interpretation of a simple indirect effect is no longer valid. The analysis must then focus on Conditional Indirect Effects (i.e., how the mediation effect changes at different levels of the moderator, which here appears to be the mediator). Please elaborate on this analysis.

*R. Thank you for your comment. If we were using the traditional methods of mediation (i.e Baron and Kenny) then, yes, our indirect estimates would be biased, as traditional methods are unable to handle interactions. However, utilising the counterfactual framework allows for the decomposition and identification of Direct and Indirect Effects in the presence of a mediator-outcome interaction (ref).*

4- Did the authors run separate single-mediator models for each factor, or did they perform a parallel or serial multiple mediation analysis? If separate models were run, please justify this choice and explain how the issue of Type I error (false positives) from running multiple tests was addressed.

*R. We ran separate single-mediator models and refer to this in the discussion. We also noted that the proportion mediated should not be additive for the individual mediators due to the possible correlation between them. However, we appreciate the issues with this method. At the time we did not have access to statistical package command that allowed more than one mediator under the counterfactual framework. Stata has now incorporated this into the latest version. We are hoping to have access to this version within the next few weeks, however at the time of this response we are unable to implement this method. In terms of adjusting for multiple comparisons, all hypotheses were generated a priori based on the literature and theory.*

*Rothman KJ. No adjustments are needed for multiple comparisons. Epidemiology. 1990 Jan;1(1):43-6. PMID: 2081237.*

5- Which version of SEVR was used? Please explain if LV pressure was not used in the limitations.

*R. The original description of SEVR measurement method was first described in 1972 by Buckberg in dogs with normal coronary arteries during cardiac catheterisation. In their method the SEVR was calculated using invasive hemodynamic methods and derived from analysis of pressure curves recorded in the left ventricle and ascending aorta during cardiac catheterization. This method is cumbersome and unsuitable for clinical practice and population studies. SEVR can be accurately and non-invasively measured using the morphological analysis of the central arterial pressure waveform as we have done in our current study. This method has excellent repeatability and is well validated. Both the original and non-invasive method as used in our current study calculate the SEVR as the ratio of the diastolic pressure time index (DPTI) to the systolic pressure time index (SPTI). Importantly it is the non-invasive method of SEVR measurement, as used in our study where the majority of the published outcome cardiovascular and validation data are derived.*

*Salvi P, Baldi C, Scalise F, Grillo A, Salvi L, Tan I, De Censi L, Sorropago A, Moretti F, Sorropago G, et al. Comparison between invasive and noninvasive methods to estimate subendocardial oxygen supply and demand imbalance. J Am Heart Assoc. 2021;10:e021207. doi: 10.1161/jaha.121.021207*

*We accept that the non-invasive method does not take into account left ventricular pressure during the cardiac cycle, which can result in overestimation of SEVR. In the light of your comments we have acknowledged this in our revised manuscript.*

6- SEVR is a dynamic measure and highly sensitive, which can be influenced by the mediators themselves (e.g., fitness via 6MWD, autonomic control via RMMSD). Please confirm that the SEVR measurements were standardized (e.g., performed under the same rest/fasting conditions) and specify if blood pressure and heart rate at the time of SEVR measurement were included as time-varying covariates in the outcome model. If not, this should be discussed as a limitation.

*R. Thank you for raising this. All SEVR measurements were taken under the same rest and fasting conditions. All participants were asked to fast and refrain from caffeine and alcohol for at least eight hours and smoking for ≥4 hours prior to their study visit. Measurements were undertaken by trained research nurses in a temperature-controlled room after the participants had rested for 5 min, lying supine with their head raised to 30°. The calculation of the SEVR uses the central arterial pressure waveform and the systolic and diastolic pressure time integrals which are influenced themselves by heart rate and systolic blood pressure. Hence, we did not adjust for these covariates. In previous studies It has been widespread published practice to use the crude SEVR for these reasons and adjustment for heart rate is not recommended. The Vicorder software SEVR result provided reflects averaged values of multiple serial cardiac cycles during the measurement period further improving is reliability.*

7- The authors mentioned sex (male) in the methods once. Did the study only include male servicemen? Did you also include females? If yes, what was any sex-difference observed in mediator analysis?

*R. Yes, you are correct. In the ADVANCE Study we only included males as stated in our method section. Whist we would have ideally liked to have examined women. The UK Operational trauma database has shown that there have been less than 12 women who have sustained serious combat-related physical injuries in the recent wars in Iraq and Afghanistan of whom the vast majority were in Iraq. Hence, they would have been excluded in ADVANCE, which reflects the recent Afghanistan conflict. Furthermore, the number of women who sustained significant injuries in ADVANCE is extremely low and no more than a handful which would have made the participants potentially identifiable.*

Minor Comments:
1- Given that the mediators were assessed approximately eight years post-injury/deployment, and the outcome (SEVR) was measured later, there is a risk of reverse causality or confounding due to post-injury lifestyle changes. Please include a dedicated limitations section addressing the temporal relationship and the challenge of establishing true causality in this context

*R. In general, we view the temporal ordering as a strength rather than a limitation. Temporal ordering is considered a prerequisite for meditation and helps to alleviate reverse causality concerns. If our mediator was measured at the same time as the injury, then we would be unable to establish if the mediator had come before or after the exposure. The same for the outcome. Additionally, the temporal ordering gives more strength to the casual inference, although we are careful not to attribute our findings to any causal link.*

*However, we acknowledge that 8 years may be a longer period of time than is optimal and many changes may have happened within that time. If we had a shorter time period between exposure and mediator then we may have minimized the possibility of exposure induced mediator-outcome confounding. Controlled direct effects can still be identified and therefore have also been presented.*

.
2- Please confirm and state whether the analysis includes all 1018 servicemen mentioned, or if there was subsequent drop-out or missing data for the SEVR measurement. If the sample size is smaller for the final analysis, please state the N used in the Methods/Results.

*The original ADVANCE Cohort consisted of 1144 participants. As stated in our methods 0f the 1053 participants who returned for the first follow up (retention >90%), there was missing SEVR data for 35 participants, leaving 1018 for these analyses. Hence, for our study we examined 1018 participants from the ADVANCE Study who had both baseline and three-year follow SEVR data available This was done to provide a robust mediation model. Our numbers are clearly stated in both the method section and in table 1 and 2. Our retention rate is considered excellent for a prospective cohort study of this type. The numbers of data points used for every mediator is detailed in the methods.*

Reviewer #3: Please see the full review in the attached PDF.

Summary:
This manuscript entitled “Mediating Factors in the Relationship between Combat-related Traumatic Injury and Myocardial Blood Flow Reserve: the ADVANCE Cohort Study” investigates whether specific cardiovascular risk factors mediate the relationship between combat-related traumatic injury (CRTI) and myocardial blood flow reserve, represented by the subendocardial viability ratio (SEVR), using longitudinal data from the ADVANCE cohort. The study leverages a valuable dataset of UK servicemen (n=1,018, 504 with CRTI, of whom 140 were amputees, and 514 uninjured men, frequency-matched, by age, rank and deployment) with a follow-up duration for more than a decade after deployment. From the standpoint of public health significance, innovation, and scientific contribution, this paper represents a substantive and meaningful addition to the emerging literature on the long-term cardiovascular consequences of military trauma. The manuscript is suitable for publication pending modifications and clarifications described below:

*R. Thank you for these supportive comments.*

Significance and Impact
The primary strength of this work lies in addressing an understudied question with substantial clinical implications: why combat-injured veterans exhibit elevated cardiovascular risk even years after injury. While prior papers from the same ADVANCE cohort established associations between CRTI, adverse cardiometabolic profiles, and reduced SEVR, this study advances the field by investigating the underlying mechanisms. Understanding modifiable mediators such as visceral fat mass, heart-rate variability, and physical function is crucial for designing targeted interventions and informing long-term rehabilitation strategies for injured service members. Given the large population of veterans with blast injuries, limb loss, and related trauma, these insights could have high translational relevance for both defence health systems and civilian trauma rehabilitation. In terms of the approach, this study’s main novelty lies in its rigorous application of counterfactual mediation analysis. Prior literature linking traumatic injury to cardiovascular risk has largely been retrospective, cross-sectional, or limited to mortality outcomes. By contrast, to strengthen causal inference, this manuscript uses longitudinal measurements: baseline (~8 years post-injury) for mediators and follow-up (~3 years later) for SEVR. The approach allows the authors to quantify the extent to which specific physiological domains (autonomic balance, adiposity, inflammation, physical activity, and physical function) explain the CRTI–SEVR association. The subgroup analysis focusing on traumatic amputation is particularly innovative and important, offering mechanistic hypotheses tailored to a clinical subgroup known to be at heightened cardiovascular risk.

*R. Thank you for these encouraging comments which provides a really good synthesis of exactly what we set out to do in our manuscript and the reasons for the specific type of study we did.*

Major comments:

- As mentioned above, beyond the scientific findings put forth in this manuscript, there are

several potentially translational findings, such as designing interventions focused on HR

variability or visceral fat. The authors are encouraged to explore this aspect further and

provide additional insight into possibilities for future clinical studies.

*R. Thank you for raising this important suggestion. We have addressed this in the discussion as follows: HRV and visceral fat are important determinants of resilience and long-term health of military servicemen. Hence, interventional strategies to increase HRV and reduce visceral fat, could have important translational advantages in the recovery trajectory of combat injured servicemen. There are several plausible strategies that could be introduced to enhance post-injury recovery/rehabilitation pathways including a stronger emphasis on a healthier lifestyle (improved diet, sleep discipline and exercise) and autonomic training.(39) ADVANCE is currently designing a feasibility study examining the use of HRV biofeedback training as an intervention to improve mental and physical health in injured British servicemen*

- Although the manuscript acknowledges its limitation in unmeasured mediator-outcome

confounding, the implication is potentially substantial. Variables such as resting heart rate,

blood pressure, lipid profile, pain severity, mental health symptoms, medication use, and

sleep quality are likely to influence both the mediators (e.g., HRV, physical function) and

SEVR. Without addressing or adjusting for these, the causal interpretations of mediation

effects remain tentative. Could the authors perform sensitivity or bias analyses to quantify

the strength of this confounding?

*R. We recognise that there are limitations to our mediation model, especially with possible exposure induced mediator-outcome confounders. This of course, may bias the Indirect Effect. We also provide the Conditional Direct Effect and the proportion eliminated, which can still be identified in the presence of an exposure induced mediator-outcome confounder and has fewer assumptions than the Natural Indirect effect.*

- Despite the decent cohort size (n=1,018) and high retention rate (~89%) large proportion

of the data is considered missing (especially the ~25% missing RMSSD values). The

missing HRV data in particular could introduce significant bias, especially if missingness

differs systematically between injured and uninjured participants. This reviewer

recommends performing a multiple imputation analysis, compare imputed vs. complete case

results, and provide missing-data patterns (e.g., missing by subgroup).

*R. Multiple imputation was considered. Unfortunately, the mediate command is not supported by the multiple imputation command in Stata. We are aware that this facility may be possible in the statistical package R and hope to utilize this in the future.*

- Lack of clear justification for treating mediators independently: The mediators (VFM,

HRV, 6MWD, LT-MVPA, Hs-CRP) are physiologically interrelated. Testing them one at

a time oversimplifies the true causal structure and risks misleading conclusions. It would

make better sense to perform sequential or joint mediation models.

*R. We acknowledge that the mediators could be interrelated. We are careful not to suggest the proportion mediated is additive and address this by stating it in the discussion.*

*“Additionally, estimating mediators separately can be misleading and the proportion mediated should not be summed to get an overall proportion mediated due to possible correlation between mediators.”*

*Additionally, we present the correlation coefficients between the mediators and show than most have weak correlations with the exception of VFM and HSCRP which have a moderate correlation.*

- The reduction in SEVR is statistically significant, but the clinical significance for this

demographic (mostly young men) is not clearly contextualized. It would be helpful if the

authors compared observed SEVR differences to established risk thresholds or known

effect sizes in metabolic syndrome, hypertension, or coronary disease cohorts.

*R. Thank you for this comment. In the discussion we have provided some additional context with references as follows: Whist the SEVR readings in our study were generally in the normal range, they were 13 units lower in the injured amputees which is an effect size not dissimilar to that observed in a recent study of metabolic syndrome among healthy adults and previously among patients with hypertension, coronary artery disease (and its severity) and diabetes mellitus compared with controls.(11, 29, 30)*

Minor comments:

- The terms “baseline” and “eight years post-injury” could be defined more clearly. A visual

description could also be included in figure 1.

R. *Apologies if this is not clear. It represents the start of the study measurements which was an average of 8 years after deployment/injury. We have tried to improve this clarity in the revised manuscript.*

- Figure 1 could be improved in quality and descriptiveness.

*R. We have amended to Figure to be of higher quality and hopefully more informative. If this is still insufficient we can redraw a new figure of higher resolution.*

- I suggest superimposing the scatter plot on figure 2 for better visualization.

*R. It is difficult to see how this can be done for this type of figure. The figure presented is the CDE for each gram of Visceral Fat with the corresponding Confidence Interval. We are unsure as what scatterplot would be suitable here.*

6. PLOS authors have the option to publish the peer review history of their article ([what does this mean?](https://gbr01.safelinks.protection.outlook.com/?url=https%3A%2F%2Ftrack.editorialmanager.com%2FCL0%2Fhttps%3A%252F%252Fjournals.plos.org%252Fplosone%252Fs%252Feditorial-and-peer-review-process%2523loc-peer-review-history%2F1%2F010f019a9ff67adb-424c3e26-5f40-48b3-96da-d811173fe6ec-000000%2FMdMgUZCQazgKhBDNvJSilElO9DpLMzxjy9yc6KkYpbA%3D238&data=05%7C02%7Cchristopher.boos%40nhs.net%7Cafe9c00a151d4d4ff3d008de284f37cc%7C37c354b285b047f5b22207b48d774ee3%7C0%7C1%7C638992518142448137%7CUnknown%7CTWFpbGZsb3d8eyJFbXB0eU1hcGkiOnRydWUsIlYiOiIwLjAuMDAwMCIsIlAiOiJXaW4zMiIsIkFOIjoiTWFpbCIsIldUIjoyfQ%3D%3D%7C0%7C%7C%7C&sdata=xCUTXGyYtFwK%2F9RxLrlwda8bUC3wqPnkCqOLKHNGHCk%3D&reserved=0)). If published, this will include your full peer review and any attached files.


If you choose “no”, your identity will remain anonymous but your review may still be made public.


**Do you want your identity to be public for this peer review?** For information about this choice, including consent withdrawal, please see our [Privacy Policy](https://gbr01.safelinks.protection.outlook.com/?url=https%3A%2F%2Ftrack.editorialmanager.com%2FCL0%2Fhttps%3A%252F%252Fwww.plos.org%252Fprivacy-policy%2F1%2F010f019a9ff67adb-424c3e26-5f40-48b3-96da-d811173fe6ec-000000%2Ff_ubRU-1k1_ef4tv5exeRFU4kotXzg1ahAScev37P_4%3D238&data=05%7C02%7Cchristopher.boos%40nhs.net%7Cafe9c00a151d4d4ff3d008de284f37cc%7C37c354b285b047f5b22207b48d774ee3%7C0%7C1%7C638992518142485124%7CUnknown%7CTWFpbGZsb3d8eyJFbXB0eU1hcGkiOnRydWUsIlYiOiIwLjAuMDAwMCIsIlAiOiJXaW4zMiIsIkFOIjoiTWFpbCIsIldUIjoyfQ%3D%3D%7C0%7C%7C%7C&sdata=QsfJBngqBYfUTZbfFa5KIHtOoQPy0YdB6thNpQTM3Ss%3D&reserved=0).

Reviewer #1: No

Reviewer #2: **Yes:** Rashid Alavi

Reviewer #3: No

[NOTE: If reviewer comments were submitted as an attachment file, they will be attached to this email and accessible via the submission site. Please log into your account, locate the manuscript record, and check for the action link "View Attachments". If this link does not appear, there are no attachment files.]

To ensure your figures meet our technical requirements, please review our figure guidelines: https://journals.plos.org/plosone/s/figures

You may also use PLOS’s free figure tool, NAAS, to help you prepare publication quality figures: https://journals.plos.org/plosone/s/figures#loc-tools-for-figure-preparation.

NAAS will assess whether your figures meet our technical requirements by comparing each figure against our figure specifications.

*In compliance with data protection regulations, you may request that we remove your personal registration details at any time.* [*(Remove my information/details)*](https://gbr01.safelinks.protection.outlook.com/?url=https%3A%2F%2Ftrack.editorialmanager.com%2FCL0%2Fhttps%3A%252F%252Fwww.editorialmanager.com%252Fpone%252Flogin.asp%253Fa%3Dr%2F1%2F010f019a9ff67adb-424c3e26-5f40-48b3-96da-d811173fe6ec-000000%2Faf0xfa47W_IpdD-O9rC7WGjIa3OUhnEOvEc8mjQ6NVw%3D238&data=05%7C02%7Cchristopher.boos%40nhs.net%7Cafe9c00a151d4d4ff3d008de284f37cc%7C37c354b285b047f5b22207b48d774ee3%7C0%7C1%7C638992518142521216%7CUnknown%7CTWFpbGZsb3d8eyJFbXB0eU1hcGkiOnRydWUsIlYiOiIwLjAuMDAwMCIsIlAiOiJXaW4zMiIsIkFOIjoiTWFpbCIsIldUIjoyfQ%3D%3D%7C0%7C%7C%7C&sdata=xpfVemxaFX0Bpa1TSKBFOEctZRiDu%2FFR7rK1Wi7qE%2BU%3D&reserved=0)*. Please contact the publication office if you have any questions.*
